# Unveiling the Impact of BMP9 in Liver Diseases: Insights into Pathogenesis and Therapeutic Potential

**DOI:** 10.3390/biom14081013

**Published:** 2024-08-15

**Authors:** Han Chen, Ying-Yi Li, Kouki Nio, Hong Tang

**Affiliations:** 1 Center of Infectious Diseases, West China Hospital of Sichuan University, Chengdu 610041, China; chenhan19890801@wchscu.cn; 2 Laboratory of Infectious and Liver Diseases, Institute of Infectious Diseases, West China Hospital of Sichuan University, Chengdu 610041, China; 3 Department of Gastroenterology, Kanazawa University Hospital, Kanazawa 9208641, Japan; liyingyi@staff.kanazawa-u.ac.jp

**Keywords:** BMP9, BMP signaling, liver disease, liver fibrosis, liver cancer

## Abstract

Bone morphogenetic proteins (BMPs) are a group of growth factors belonging to the transforming growth factor β(TGF-β) family. While initially recognized for their role in bone formation, BMPs have emerged as significant players in liver diseases. Among BMPs with various physiological activities, this comprehensive review aims to delve into the involvement of BMP9 specifically in liver diseases and provide insights into the complex BMP signaling pathway. Through an enhanced understanding of BMP9, we anticipate the discovery of new therapeutic options and potential strategies for managing liver diseases.

## 1. Introduction

Bone morphogenetic proteins (BMPs) constitute a group of signaling molecules that belong to the transforming growth factor β(TGF-β) superfamily [1]. Their significance extends beyond their initial identification as inducers of bone formation [2], as they are now recognized for their vital roles in diverse biological processes such as embryonic development, tissue regeneration, and organ homeostasis. BMPs exert regulatory control over cell proliferation, differentiation, apoptosis, and metabolism across various tissues and organs in the body [3,4,5]. These multifaceted effects position BMPs as crucial factors in both physiological and pathological conditions [6,7,8,9].

**Figure 1:**
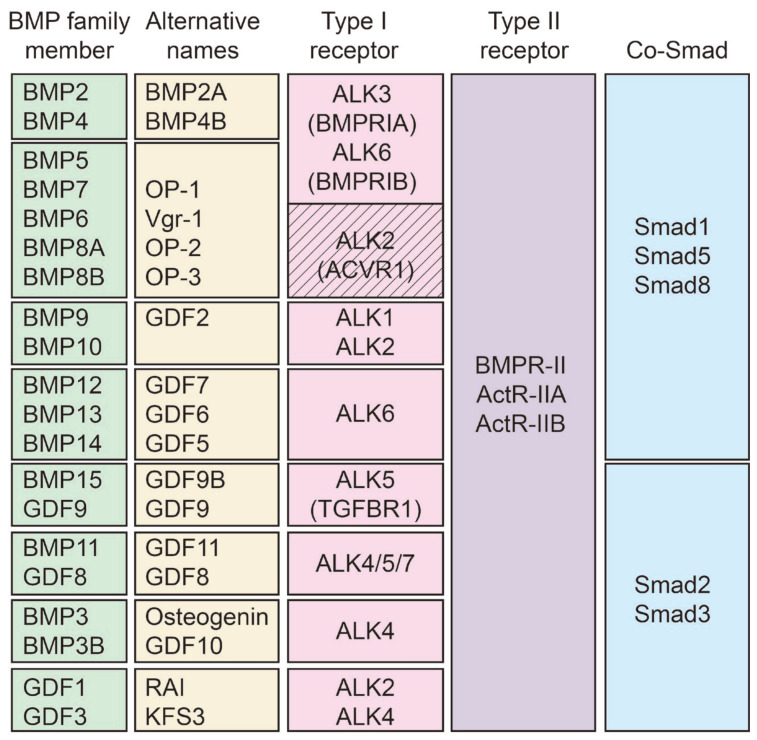
BMP proteins and receptors. The summary of BMP family members and their alternative names, type I and type II receptors, and Co-Smads. OP, osteogenic protein; Vgr, vegetal related; RAI, atrial isomerism right; KFS3, Klippel-Feil syndrome 3, autosomal dominant; ALK, anaplastic lymphoma kinase; BMPRIA, bone morphogenetic protein receptor IA; BMPRIB, bone morphogenetic protein receptor IB; ACVR1, Activin A receptor, type I.

The TGF-β superfamily comprises three subfamilies: the TGF-β subfamily, the bone morphogenetic protein and growth differentiation factor (BMP/GDF) subfamily, and the activin and inhibin subfamily. The BMP/GDF subfamily, characterized by sequence homology and functional diversity, can be further divided into distinct groups: BMP2/4, BMP5/6/7/8A/8B, BMP9/BMP10, BMP12/13/14, BMP15/GDF9, BMP11/GDF8, BMP3/BMP3B, and GDF1/3 [9,10]. These proteins, known by alternative names, interact with various type I and II receptors, phosphorylate Co-Smads downstream, translocate into the nucleus, and regulate targeted genes (Figure 1) [11,12]. Among these members, BMP9 (also known as GDF2) stands out for its predominant expression in the liver. Initially recognized for its role in hepatogenesis [13], hematopoiesis [14], osteogenesis [15], and chondrogenesis [16,17,18]. BMP9 has emerged as a regulator of hepatic glucose and lipid metabolism [19], iron balance [20], cholinergic neuron differentiation [21,22], endothelial tip/stalk cell selection [23], angiogenesis [24,25,26], lymph angiogenesis [27], liver regeneration, and fibrosis [28,29]. BMP9 is an important mediator of vascular quiescence, and the loss-of-function mutations in the BMP9—ENG—ALK1 signaling pathway lead to hereditary hemorrhagic telangiectasia (HHT), an inherited disorder characterized by vascular dysplasia and potential bleeding tendencies [30,31]. Furthermore, BMP9 deficiency is associated with pulmonary arterial hypertension (PAH), a progressive obliterative vasculopathy affecting the distal pulmonary arterial circulation [30,32,33,34]. Additionally, BMP9 has been implicated in liver diseases, including nonalcoholic fatty liver disease (NAFLD), liver fibrosis, and hepatocellular carcinoma(HCC) [35]. However, many aspects regarding its precise involvement and underlying mechanisms in these conditions remain elusive [36]. This review aims to provide a comprehensive overview of BMP9’s roles and mechanisms in liver diseases. By examining its involvement, we can explore potential therapeutic options for the treatment of liver diseases.

## 2. Receptors and Intracellular Signaling of BMP9

**Figure 2:**
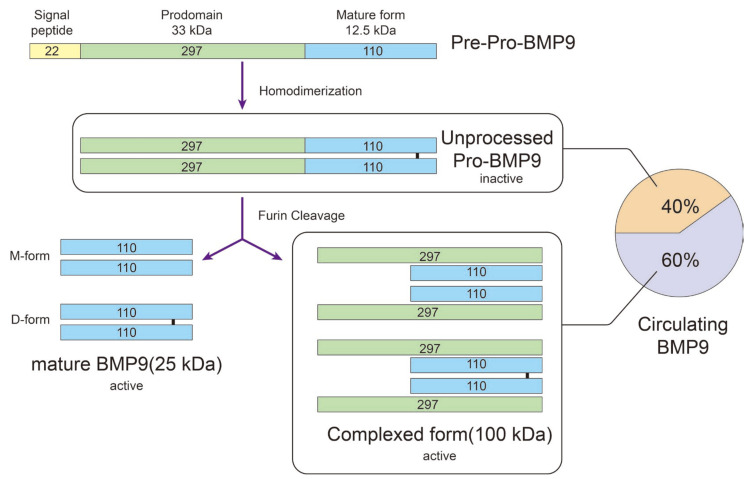
Synthesis procedure for BMP9. BMP9 is synthesized as a 429 amino acid (aa) precursor protein known as pre–pro-BMP9. It consists of a 22 aa signal peptide, a 297 aa prodomain, and a 110 aa mature protein. After synthesis, the pre-pro-BMP9 undergoes homodimerization and subsequent cleavage, resulting in two active forms: the short mature BMP9 (25 kDa) and the complexed form (100 kDa). The short mature BMP9 (25 kDa) includes two forms of dimers, with (D-form) or without (M-form) an intermolecular disulfide bond. Within circulating BMP9, the inactive unprocessed pro-BMP9 accounts for 40%, while the active complexed form accounts for 60%.

The BMP9 protein, which is encoded by the *GDF2* gene, is located at 10q11.22. BMP9 is initially synthesized as a precursor protein called pre-pro-BMP9, which consists of a total of 429 amino acids (aa). This includes a signal peptide (22 aa), a prodomain (297 aa), and a mature protein region (110 aa). The pre-pro-BMP9 then homodimerizes to form unprocessed pro-BMP9, which remains in an inactive state [37,38]. The processing of BMP typically occurs intracellularly within the Golgi apparatus. After being cleaved by a serine endoprotease, two active forms of BMP9 are synthesized. These include the short mature form (25 kDa) and the complexed form (100 kDa), both of which result from the Furin cleavage of pre-pro-BMP9 [37,38]. The stable BMP9 dimers can either form with an intermolecular disulfide bond (D-form) or without it (M-form). By changing the redox potential, these two forms can be converted into each other, leading to a significant alteration in BMP9 stability. The M-form is more susceptible to redox-dependent cleavage by proteases found in serum [39]. In both humans and mice, biologically active concentrations of BMP9 can be detected in the circulation. The circulating forms of BMP9 comprise the active complexed form of BMP9 (60%) and the inactive pro-BMP9 (40%), with both forms exceeding 100 kDa in size (Figure 2) [40,41]. It has been reported that BMP9 and BMP10 in plasma can form heterodimers, which contribute to the majority of the biological BMP activity [42]. Currently, commercial kits utilizing the Enzyme-Linked Immuno-Sorbent Assay (ELISA) are available for detecting various forms of BMP9 in serum. Some antibodies specifically target the mature form of BMP9, displacing the cleaved prodomain from the BMP9 complexed form. As a result, this type of ELISA quantifies mature BMP9 regardless of its association with the prodomain. However, it does not recognize the unprocessed BMP9 precursor. Alternatively, a combination of anti-prodomain BMP9 and anti-mature BMP9 antibodies as capture and detection antibodies, respectively, has proven effective in detecting the unprocessed forms of BMP9 [43].

BMP9 exerts its biological functions by binding to specific receptors. BMP receptors form heterotetrameric complexes, consisting of two BMP type I receptors and two type II receptors. The BMP type I receptors include ALK1, ALK2, ALK3 (BMPRIA), and ALK6 (BMPRIB), while the BMP type II receptors consist of BMPR-II, ActR-IIA, and ActR-IIB (Figure 1) [33,44]. BMP9 exhibits sub-nanomolar affinities (EC50 of around 50 pg/mL) when binding to the type I receptor ALK1 [45]. Both mature BMP9 and the BMP9 complexed form bind to ALK1 with the same level of affinity. Both the D-form and M-form of mature BMP9 are capable of binding to the prodomain and ALK1 [39]. However, BMP9’s affinity for ALK2 is significantly lower compared to ALK1. Additionally, the BMP9 complexed form has an even lower affinity for ALK2 than the mature BMP9. Regarding the type II receptors, some studies suggest that BMP9 binds with similar affinities to ALK1 and ActR-IIB, while showing lower affinities towards BMPRII and even lower affinities towards ActR-IIA [31,37,38,45]. Nevertheless, contradictory findings suggest that BMP9 has a much higher affinity for ALK1 than for the type II receptors, correlating with their expression levels in endothelial cells. Moreover, it has been observed that the binding of BMP9 to ALK2 is greatly facilitated when BMPR-II or ActR-IIA/B receptors are co-expressed, indicating that in such cases, binding to the type II receptors precedes binding to the type I receptor. Also, even in the absence of ALK1, cells are still capable of responding to BMP9 through ALK2 [46,47]. The expression of ALK2 and ActR-IIA was detected in whole human hepatic tissue, while BMPR-II expression was observed in primary mouse hepatocytes and HCC cell lines [48]. ALK1 was found to be expressed on the hepatoma cell line HLE [49]. It is intriguing to analyze the expression patterns of BMP9 receptors in various HCC cells, as this can provide valuable insights into the mechanisms by which BMP9 influences HCC [35].

**Figure 3:**
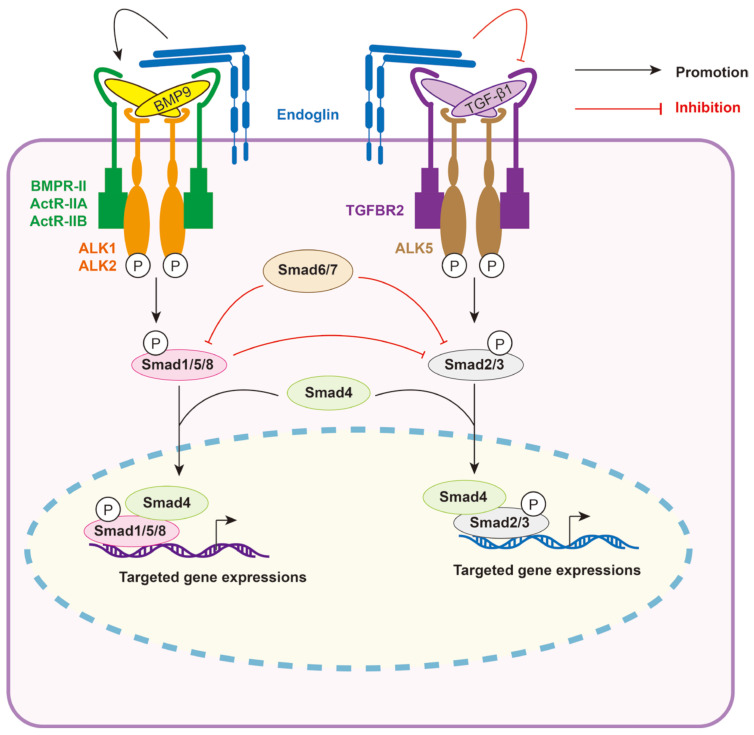
Diagram of TGF-β and BMP9 signaling pathways. BMP9 binds to heterotetrameric receptor complexes (ALK1/ALK2 and BMPR-II/ActR-IIA/ActR-IIB), resulting in the phosphorylation of downstream Smad1/5/8 (pSmad1/5/8). pSmad1/5/8 subsequently interacts with Smad4, translocates into the nucleus, and binds to the promoters of target genes, thereby promoting gene expression. The co-receptor endoglin plays a crucial role in activating the BMP9—pSmad1/5/8 pathway. Similarly, TGF-β binds to heterotetrameric receptor complexes (ALK5 and TGFBR2), leading to the phosphorylation of downstream Smad2/3 (pSmad2/3). pSmad2/3 then interacts with Smad4, translocates into the nucleus, and binds to the promoters of target genes, thereby promoting gene expression. pSmad1/5/8 are negative regulators of Smad3-dependent gene transcription. Smad6 and Smad7 inhibit the phosphorylation of Smad1/5/8 and Smad2/3.

BMP9 exhibits high affinity direct binding to the co-receptor endoglin (ENG), which is highly expressed in endothelial cells [50,51,52]. Endoglin enhances the signaling output, as evidenced by increased levels of pSmad1/5/8 [53,54,55]. The binding of BMP9 to the type II receptors leads to the displacement of the prodomain and cell surface endoglin, resulting in the formation of a signaling complex [31,52]. Furthermore, soluble endoglin can be detected in the bloodstream and has traditionally been believed to function as a ligand trap for circulating BMP9 [51]. In parallel, upon binding of TGF-β1, ALK5/TGFBR2 stimulation leads to the phosphorylation of Smad2/3. pSmad2/3 subsequently interacts with Smad4, facilitating its translocation to the nucleus to regulate multiple gene expressions [56]. It should be noted that Smad3 has the ability to directly bind to Smad-binding elements found within gene promoters, leading to an enhancement of transcription. On the other hand, neither Smad2 nor Smad4 possess DNA binding domains. Instead, they function as regulators of Smad3-mediated gene transcription [57,58,59,60]. pSmad1/5/8 could suppress Smad3-dependent gene transcription, and this may be the reason why BMP9—pSmad1/5/8 signaling counteracts TGF-β1—pSmad2/3 signaling [61,62,63,64]. The I-Smads (or Inhibitory Smads) Smad6 and Smad7 inhibit the phosphorylation of Smad1/5/8 and Smad2/3 (Figure 3) [65,66].

**Figure 4:**
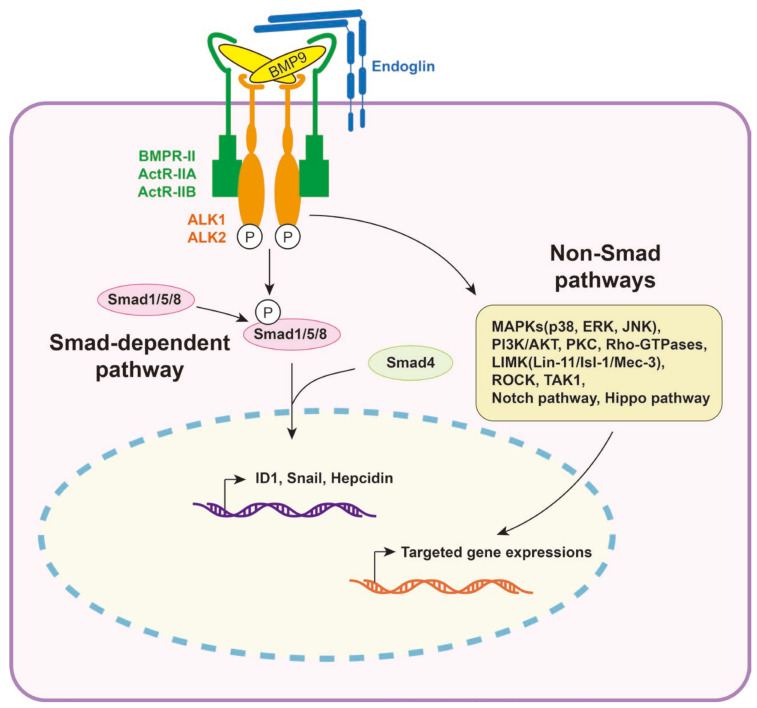
Smad-dependent pathway and non-Smad pathways.

Upon binding of BMP9, the type II receptor initiates the phosphorylation of the type I receptor, leading to the subsequent phosphorylation of Smad1/5/8 within 5 min. Compared to the D-form of mature BMP9, the M-form shows a lesser degree of sustained induction in Smad1/5/8 phosphorylation [39]. The duration of this phosphorylation period depends on the concentration of BMP9, ranging from a few hours with low doses (0.1 ng/mL) to up to 24 h with higher doses (10 ng/mL) [53,67]. The BMP9/ALK1 complex undergoes rapid endocytosis and can either be recycled back to the membrane or degraded through proteolysis [68]. Additionally, it has been observed that in endothelial cells, BMP9 can induce the phosphorylation of Smad2, although at a much lower level compared to Smad1/5/8 phosphorylation [31,69,70]. Apart from the well-studied canonical Smad-dependent pathway, significant research has been conducted on the non-canonical non-Smad BMP pathways. The downstream effects of BMP9 involve various signaling molecules such as mitogen-activated protein kinases (MAPKs, including p38, extracellular-signal-regulated kinase (ERK), and c-Jun N-terminal kinases (JNK)), Phosphoinositide 3-kinases/AKT (PI3K/AKT), LIM Domain Kinase (LIMK), Rho, Rho-Associated Coiled-Coil-Containing Protein Kinase (ROCK), and TAK1 [71,72,73]. Additionally, there are crucial interactions between Notch, VEGF, Hippo, EGF, and BMP9 [74,75,76,77]. Some of the most prominent target genes of BMP9 include ID1, ID2, and ID3 [78,79], along with other notable targets such as Hepcidin and Snail (Figure 4) [35,80,81,82].

The regulation of BMP9 involves several processes, including transcription, microRNA, and ligand traps. A study on acetaminophen (APAP)-induced acute liver injury (ALI) demonstrated that BMP9 expression is regulated by CCAAT/enhancer binding protein α (C/EBPα), both in vitro and in vivo [83]. Additionally, miR-761 has been identified as a targeting inhibitor of BMP9 [84]. Furthermore, Crossveinless 2 (CV2), soluble ENG (sENG), and soluble chimeric ALK1 protein (ALK1-Fc) have been considered potential ligand traps for BMP9 [41,52,85]. However, our understanding of BMP9 regulation remains incomplete, and further studies are needed to elucidate the regulators and regulatory mechanisms involved.

## 3. BMP9 and Liver Cell Biology

In both humans and mice, BMP9 is predominantly expressed in the liver and originates from various cell types including hepatocytes, cholangiocytes, and hepatic stellate cells (HSCs), among which intrahepatic biliary epithelial cells express the highest levels of BMP9 [41]. Furthermore, BMP9 expression is observed to increase upon HSC activation [28,86]. In rats, however, BMP9 is primarily produced by HSCs, Kupffer cells (KCs), and endothelial cells [87]. Interestingly, studies have shown a higher expression of BMP9 in aged mice compared to their younger counterparts [83].

BMP9 plays a crucial regulatory role in liver cell functions. In healthy liver, it has the ability to inhibit hepatocyte proliferation and epithelial-to-mesenchymal transition (EMT) while preserving the expression of important metabolic enzymes such as cytochrome P450 [28]. Furthermore, BMP9 is involved in the maturation and differentiation of hepatic progenitor cells [88]. It facilitates the functioning of these cells, which are responsible for repopulating the liver and restoring its functionality after severe damage, through the HGF/c-Met signaling pathway [88,89]. The expression of BMP9 in Dissè’s spaces indicates its regenerative role in the liver [29]. In terms of oval cells, BMP9 directly inhibits their expansion through the ALK2—pSmad1/5/8 pathway, as ALK2 expression is significantly higher than that of ALK1 in oval cells [90]. The BMP9/BMP10—ALK1 signaling pathway controls the identity and self-renewal of Kupffer cells (KCs) through a Smad4-dependent pathway. The development of Kupffer cells crucially depends on their crosstalk with hepatic stellate cells via the evolutionarily conserved ALK1-BMP9/10 axis [91]. However, it should be noted that ALK1 is not essential for the maintenance of macrophages located in the lung, kidney, spleen, and brain [92]. The loss of BMP9 in the 129/Ola genetic background leads to spontaneous liver fibrosis due to the capillarization of LSEC and kidney lesions [93,94]. Furthermore, hepatic endothelial ALK1 signaling protects against the development of vascular malformations, preserving organ-specific endothelial differentiation and angiocrine signaling. On the other hand, the loss of ALK1 causes hepatic vascular malformations [95].

## 4. BMP9 and Viral Hepatitis

Currently, there have been reports indicating an inverse correlation between disease stage and circulating levels of BMP9 in patients with hepatitis C virus (HCV) [96]. However, there is a lack of available references directly addressing the effects of BMP9 on viral hepatitis. Nonetheless, several studies have investigated the interactions of other BMPs with hepatic viral infections. It has been observed that there exists a mutual antagonistic relationship between HCV infection and the BMP—Smad pathway. HCV infection attenuates the induction of hepcidin expression by BMP6, possibly through the TNF-mediated downregulation of the BMP co-receptor hemojuvelin. Conversely, BMP—Smad activity influences antiviral immunity. BMP6 regulates a gene profile reminiscent of type I interferon (IFN) signaling, including the upregulation of interferon regulatory factors (IRFs) and the downregulation of an inhibitor factor of IFN signaling called Ubiquitin specific peptidase 18 (USP18). BMP6 enhances the transcriptional and antiviral responses to IFN while also effectively inhibiting HCV replication independently of IFN, along with related activin proteins. Furthermore, both BMP6 and activin A have been found to suppress the growth of hepatitis B virus (HBV) in cell culture [97]. However, it is important to note that the current studies on the interactions between BMPs and viral hepatitis are limited. Furthermore, BMP9 shows relatively low homology with BMP6. It is not advisable to speculate on the mutual interaction between BMP9 and viral hepatitis based on the interaction between BMP6 and viral hepatitis. Therefore, further research is necessary to fully understand and explore the potential associations and implications of BMP9 in the context of viral hepatitis.

## 5. BMP9 and Acute Liver Injury

Acute liver injury induced by partial hepatectomy, as well as single injections of carbon tetrachloride (CCl_4_) or lipopolysaccharide (LPS) in mice, leads to a temporary decrease in hepatic BMP9 mRNA expression. Interestingly, the application of BMP9 following partial hepatectomy has been shown to significantly aggravate liver damage and disrupt the regenerative response [28]. However, the underlying mechanism remains unclear and requires further investigation. Notably, it has been discovered that LPS triggers the secretion of factors such as IL-6 from human liver sinusoidal endothelial cells (LSECs), which, in turn, upregulate BMP9 expression in human liver myofibroblasts [86]. Another intriguing finding is that aging-related C/EBPα plays a role in upregulating BMP9 expression, contributing to the exacerbation of APAP-induced ALI associated with aging. Firstly, BMP9 directly increases the susceptibility of hepatocytes to APAP-induced cell death. Secondly, it contributes to the polarization of M1 macrophages. These results highlight the significant impact of aging-associated C/EBPα on BMP9 expression, leading to the deterioration of APAP-ALI during the aging process [83]. Furthermore, in a model of cholestatic liver injury induced by 3,5-diethoxicarbonyl-1,4-dihydrocollidine (DDC), BMP9 has been identified as a negative regulator of oval cell expansion. The deletion of BMP9 in mice reduces liver damage and fibrosis but intensifies inflammation upon DDC feeding through the activation of the PI3K—AKT, ERK—MAPK, and c-Met signaling pathways. Additionally, due to higher expression of ALK2 compared to ALK1 in oval cells, BMP9 inhibits oval cell expansion via the ALK2—p-Smad1/5/8 pathway [90]. These findings collectively suggest that BMP9 is downregulated during acute or chronic liver injury caused by hepatotoxic substances or partial surgical excision. In such scenarios, elevated levels of BMP9 may further exacerbate liver damage.

## 6. BMP9 and NAFLD

### 6.1. BMP9 in Lipid Metabolism and Obesity

The beneficial effects of BMP9 on NAFLD are evident through its negative correlation with liver steatosis. A cross-sectional study observed that patients with both type 2 diabetes mellitus (T2DM) and NAFLD had significantly lower levels of serum BMP9 [98]. Furthermore, the study identified independent factors, such as fasting insulin (FINS), low-density lipoprotein cholesterol (LDL-C), high-density lipoprotein cholesterol (HDL-C), and body mass index (BMI), that influenced serum BMP9 levels. Among the fibroblast growth factor (FGF) family, FGF19 plays a crucial role in regulating glucose and lipid metabolisms [99]. Considering this, increased hepatic BMP9 levels would likely be beneficial, especially in pre-steatotic conditions, as BMP9 directly induces FGF19 in the gut [100]. Similarly to FGF19, FGF21 also plays a critical role in regulating glucose and lipid metabolisms. Animal studies have demonstrated that FGF21 can improve insulin sensitivity in obese animals, reduce body weight, lower the levels of low-density lipoprotein cholesterol, and reverse hepatic steatosis [101,102]. By enhancing FGF21 expression, MB109 (a recombinant derivative of human BMP9) effectively reduces obesity-induced liver pathologies, including inhibiting lipid accumulation in the liver and significantly decreasing serum levels of alanine aminotransferase (ALT), aspartate transaminase (AST), and total cholesterol. Additionally, MB109 exhibits dose-dependent improvements in the glucose and lipid metabolisms of obese mice. However, it is important to note that insufficient doses of MB109 may not adequately address the aforementioned pathological manifestations [103]. BMP9 has also been shown to suppress NAFLD, reduce obesity, improve glucose metabolism, alleviate hepatic steatosis, and decrease inflammation by reducing the promoter chromatin accessibility of Cers6, Fabp4, Fos, and Tlr1 [104]. Another study involving both animal models and cell lines demonstrated that BMP9 ablation promotes liver steatosis, with the mechanism being that BMP9 treatment attenuates triglyceride accumulation by enhancing peroxisome proliferator-activated receptor α (PPARα) promoter activity through the activation of phosphorylated Smad (pSmad) [105].

Adipose tissue plays a crucial role in maintaining energy balance and presents a potential target in the fight against obesity [106]. It is closely associated with energy homeostasis and offers an opportunity to address obesity, insulin resistance (IR), and T2DM. There are two distinct types of adipose tissue: white adipose tissue (WAT) and brown adipose tissue (BAT). WAT primarily serves as a lipid reservoir, while BAT dissipates energy through thermogenesis, generating heat. Furthermore, WAT functions as an endocrine organ by releasing adipokines. In cases of obesity, dysfunctional WAT has been linked to the secretion of pro-inflammatory adipokines, leading to IR, T2DM, and NAFLD/nonalcoholic steatohepatitis (NASH) [107]. On the other hand, BAT has been shown to regulate glucose homeostasis and improve insulin sensitivity. Therefore, inducing Adipose Tissue Browning is considered a strategy to combat obesity [106,108].

It has been discovered that BMP9 plays a role in activating thermogenesis in BAT and promoting the “browning” process in WAT [109]. Consequently, it has gained recognition as a potent inducer of brown adipogenesis with significant potential in combating obesity [110]. Moreover, when exposed to cold temperatures, BMP9 expression is triggered by the binding of cAMP response-element binding protein (CREB) and CREB-binding protein (CBP) to the BMP9 promoter. This activation leads to the stimulation of the Smad1 and MAPK pathways, which subsequently induce the expressions of uncoupling protein 1 (UCP1) and peroxisome proliferator-activated receptor-gamma coactivator 1 alpha (PGC1α), ultimately facilitating the “browning” of white adipose tissue. These findings underscore the substantial therapeutic value of BMP9 in addressing obesity-related NAFLD.

However, a cross-sectional study found a positive correlation between the serum levels of BMP9 and NAFLD/NASH. Specifically, patients with BMP9 levels greater than 1188 pg/mL had more severe NAFLD/NASH, suggesting that BMP9 could serve as a potential biomarker for these conditions [96]. In animal studies, BMP9 promoted nonalcoholic steatohepatitis by polarizing M1 macrophages through enhancing NF-κB signaling activation [111]. The conflicting results may be attributed to the small sample sizes and the use of different animal models in these clinical studies. Thus, future research should focus on precise and large-scale clinical studies with well-designed protocols, as well as comprehensive animal experiments, to better understand the regulatory role of BMP9 in NAFLD. Endoglin is a target of BMP9. An animal model demonstrated that mice fed with a high fat, fructose, and cholesterol (FFC) diet showed elevated serum and hepatic endoglin expression levels. Elevated levels of human serum endoglin lead to increased cholesterol deposition in the liver due to reduced conversion into bile acids. This also alters triglyceride (TAG) metabolism, causing their accumulation in the liver. This occurs through a combination of decreased TAG elimination via β-oxidation and reduced hepatic efflux of TAG. Elevated soluble endoglin (sEng) levels may contribute to the progression of NASH by compromising the crucial protective mechanism that prevents the excessive accumulation of TAG and cholesterol in the liver. These findings emphasize the significance of high serum endoglin levels in individuals susceptible to developing NASH [112].

### 6.2. BMP9 in Glucose Metabolism and T2DM

T2DM is a significant risk factor for NAFLD, and there is a strong association between these conditions [113,114]. The prevalence of NAFLD in patients with T2DM exceeds 60% [115]. In individuals with T2DM, impaired insulin regulation leads to increased liver fat accumulation, contributing to the development of NAFLD. NAFLD, in turn, worsens insulin resistance, creating a detrimental cycle between the two conditions [116]. Therefore, addressing these interconnected conditions and adopting a holistic approach to treating metabolic diseases could be highly beneficial [117].

Studies have reported a negative association between circulating BMP9 and metabolic syndrome as well as insulin resistance [118]. Circulating BMP9 levels were significantly lower in newly diagnosed T2DM patients compared to healthy subjects. BMP9 levels negatively correlated with HbA1c, fasting blood glucose, oral glucose tolerance tests (OGTT), the area under the curve for glucose (AUC glucose), and the homoeostasis model assessment of insulin resistance (HOMA-IR). Thus, the decreased levels of circulating BMP9 in type 2 diabetes patients serve as a marker of insulin resistance [119]. BMP9 has a protective effect against insulin resistance. Platycodins induce the activation of the BMP9/Smad4 pathway, which corrects blood glucose and lipid metabolism disorders, improves the liver index, and protects the liver function in cases of liver complications related to type 2 diabetes [120]. Conversely, the deletion of BMP9 elevated the phosphoenolpyruvate carboxykinase (PEPCK) protein levels and lowered the levels of InsR and Akt phosphorylation in mouse models fed a high-fat diet, resulting in exacerbated insulin resistance and glucose intolerance [121]. Additionally, BMP9 deletion in non-diabetic rats leads to insulin resistance and glucose intolerance [122]. BMP9 facilitates insulin action by reducing phosphoenolpyruvate carboxykinase expression, inhibiting hepatic glucose production, and increasing glycogen synthesis through the activation of the Smad5—Akt2—glycogen synthase kinase 3 (GSK3) pathway in L6 myotubes [123,124]. From the above research, we can infer that BMP9 possesses insulin-like properties. Therefore, BMP9 could be a potential candidate for enhancing hepatic insulin sensitivity. In conclusion, BMP9 plays a positive role in insulin resistance and T2DM, and it has the potential to become a new target for the treatment of NAFLD (Table 1).

## 7. BMP9 and Hepatic Fibrosis

Hepatic fibrosis refers to the excessive accumulation of collagen and other extracellular matrix components in the liver. It is a progressive condition that can result from chronic liver injury, including viral hepatitis, alcohol abuse, NAFLD, autoimmune liver diseases, or prolonged exposure to toxins. While the liver has an impressive regenerative capacity, continuous or severe injury can trigger an abnormal wound healing response [127,128,129]. This response involves the activation of HSCs, which are responsible for producing and depositing collagen and other connective tissue proteins [130,131]. Over time, this deposition leads to the formation of scar tissue, impairing liver function and disrupting its normal architecture. As hepatic fibrosis progresses, it can develop into a more advanced stage known as cirrhosis. Cirrhosis is characterized by extensive fibrotic scarring, nodules, and distortion of the liver structure [132]. It can lead to complications such as portal hypertension, impaired liver function, increased risk of liver cancer, and ultimately, liver failure [133]. Considerable research has been conducted to investigate the impact of BMP9, its receptors, and targets on liver fibrosis [81]. BMP9 has been found to have a protective effect on liver fibrosis by controlling the fenestration of liver sinusoidal endothelial cells and modulating the signaling of secreted cytokines, thus attenuating hepatic fibrosis [134]. In mouse models, the deletion of BMP9 resulted in liver fibrosis, specifically in the 129/Ola background [94]. However, it has also been observed that serum BMP9 levels are significantly higher in patients with fibrosis compared to healthy individuals (*p* < 0.01) [134]. Therefore, further extensive research is needed to clarify whether BMP9 promotes or prevents hepatic fibrosis, as well as to elucidate the specific mechanisms involved. Given that the BMP9 receptors ALK1 and Endoglin, as well as the BMP9 downstream targets Hepcidin and ID1, have significant roles in liver fibrosis, we will comprehensively discuss each of these components individually.

### 7.1. BMP9–ALK1 Axis in Liver Fibrosis

The activation of liver non-parenchymal cells plays a crucial role in the process of liver fibrosis. Among these cells, ALK1 is the primary receptor for BMP9 in the liver [35]. When ligands bind to ALK1, it triggers the phosphorylation of Smad1, which subsequently promotes the expression of ID1. This induction leads to the differentiation of HSCs into fibroblasts, contributing to the production of extracellular matrix (ECM) proteins [64,135]. In the mouse liver, HSCs serve as both the source and target of BMP9. A deficiency of BMP9 or the overexpression of the selective BMP9 antagonist ALK1-Fc has been found to reduce collagen accumulation and subsequent fibrosis in cases of chronic liver damage in mice [28]. Furthermore, an herbal compound called Cpd861 has shown potential in inhibiting the activation of LX-2 cells by suppressing the TGF-β1–ALK1–Smad1 pathway, thereby restraining the expression of alpha smooth muscle actin (α-SMA). This inhibition was also observed in rats with liver fibrosis induced by bile duct ligation (BDL), where Cpd861 induced the expression of the SnoN protein and inhibited the TGF-β1–Smad signaling pathway, resulting in the attenuation of liver fibrosis [136]. The ectopic expression of BMP9 has been found to exacerbate liver fibrosis in mouse models induced by CCl_4_ and BDL. BMP9 activates the ALK1–Smad1/5/8–ID1 pathway, promoting the activation of HSCs during the progression of liver fibrosis. Conversely, inhibiting BMP9 expression or neutralizing BMP9 protein using anti-BMP9 monoclonal antibodies could alleviate hepatic fibrosis [134]. Therefore, there is a need to further elucidate the role and molecular mechanisms of the BMP9–ALK1 axis in regulating hepatic fibrosis, which could have significant clinical and biological implications.

### 7.2. BMP9–Endoglin Axis in Liver Fibrosis

Endoglin is a co-receptor for both TGF-β and BMPs. While it cannot bind ligands on its own, it can interact with BMP9 in the presence of type I or type II signaling receptors. Endoglin is expressed in various cell types including endothelial cells, epithelial cells, fibroblasts, septal myofibroblasts, and HSCs. The upregulation of endoglin has been commonly observed in liver injuries, suggesting its potential as a serum biomarker for liver fibrosis. In a study of pediatric cholestatic liver disease, endoglin, along with interleukin-8 (IL-8) and matrix metalloproteinase-7 (MMP-7), showed a significant correlation with increased liver stiffness in children with biliary atresia [137]. Additionally, patients with alcoholic liver cirrhosis exhibited significantly decreased concentrations of endoglin, zinc, and selenium in their serum. However, patients with liver cirrhosis and carcinoma had elevated levels of endoglin in their serum [138,139]. Patients with HCV-related cirrhosis also demonstrated higher levels of serum endoglin compared to those with fibrosis but without cirrhosis [140]. Furthermore, in patients with advanced HCV-related fibrosis, intrahepatic expression of endoglin was significantly higher than in patients with early fibrosis and normal liver [138,140]. HCV infection can induce liver pathogenesis by increasing endoglin expression and activating the ALK1–Smad1/5 pathway [141]. A specific variant of endoglin, Thr5Met, has been associated with an increased risk of fibrosis development in HCV carriers [142]. Endoglin is involved in fibrogenic Smad signaling [138,143]. It regulates TGF-β and BMP signals in liver fibrosis by inhibiting the ALK5–Smad2/3 pathway and enhancing the ALK1–Smad1/5 pathway [144]. In a mouse model, different isoforms of endoglin were found to be upregulated in liver samples from patients with chronic and acute liver injury. Notably, a deficiency of endoglin in HSCs significantly exacerbated fibrosis, suggesting that endoglin may play a protective role against fibrotic injury through the modulation of TGF-β signaling [145]. In conclusion, endoglin plays a role in fibrogenic Smad signaling and regulates TGF-β and BMP signals in liver fibrosis by modulating the ALK5–Smad2/3 pathway and enhancing the ALK1–Smad1/5 pathway. Further studies are needed to fully understand the role of the BMP9–endoglin axis in liver fibrosis and its underlying mechanisms.

### 7.3. BMP9–Hepcidin Axis in Liver Fibrosis

Iron deposition in the liver is recognized as a critical factor in the development and progression of liver disease [146]. Hepcidin, a peptide hormone primarily synthesized and secreted by the liver, plays a crucial role in regulating iron metabolism in the body. It acts as a key regulator of systemic iron homeostasis by controlling iron absorption, recycling, and storage. BMP ligands can regulate the hepcidin levels through BMP receptors and the co-receptor hemojuvelin with the exception of BMP9, which does not interact functionally with hemojuvelin [147]. BMP9 has been shown to upregulate hepcidin expression through SMAD1/5 signaling. In a mouse model, hepatocytes lacking Smad1/5/8 exhibited severe tissue iron loading and liver fibrosis [148]. A meta-analysis of several studies revealed that hepcidin levels were significantly lower in subjects with HCV infection but higher in subjects with NAFLD. No significant differences in hepcidin levels were observed in subjects with HBV infection or controls. Furthermore, patients with cirrhosis of any etiology generally had lower hepcidin levels [149,150,151,152]. However, a cross-sectional study including treatment-naïve patients with chronic hepatitis B (CHB) indicated that reduced hepcidin levels were associated with increased fibrosis severity and histological activity index (HAI) in CHB patients. In autoimmune liver diseases, such as autoimmune hepatitis (AIH) and primary biliary cholangitis/primary sclerosing cholangitis (PBC/PSC), both serum hepcidin levels and the serum hepcidin/ferritin ratio were significantly lower compared to HBV, HCV, or NAFLD cases. These levels also correlated negatively with serum alkaline phosphatase (ALP) levels. PBC/PSC and AIH patients maintained low serum hepcidin throughout their two-year treatment period [153]. These findings suggest that hepcidin may play a protective role in liver fibrosis. Interestingly, the absence of hepcidin expression inhibits hepatic lipid accumulation caused by a high-fat diet, but it can also lead to the early development of fibrosis [154]. Conversely, the overexpression of hepcidin has been shown to alleviate steatohepatitis and fibrosis in a mouse model of diet-induced nonalcoholic steatohepatitis [155]. In summary, hepcidin levels are closely associated with liver fibrosis, but whether they are increased or decreased depends on the specific etiology. Further research is needed to fully understand the complex role of hepcidin in liver fibrosis and its underlying mechanisms.

### 7.4. BMP9-ID1 Axis in Liver Fibrosis

ID1 is a direct target of BMP9 [156] and plays a crucial role in hepatic fibrogenesis. It is interesting that in HSCs, ID1 could be induced by TGF-β1 through the ALK1–Smad1 pathway but not by the ALK5–Smad2/3 pathway and knockdown of ID1 in HSCs interfered with α-SMA fiber formation [157]. This indicates that ID1 may be a protective factor for liver fibrosis. ID1 has been identified as a potential target for therapeutic interventions of liver fibrosis. Ursodeoxycholic acid (UDCA), for instance, can alleviate liver fibrosis by promoting liver regeneration through the activation of the ID1-Wnt Family Member 2 (WNT2)/hepatocyte growth factor (HGF) pathway. The protective effects of UDCA were diminished when ID1 was knocked down in BDL mice. In a CCl_4_-induced liver fibrosis model, the deletion of Smad4 in hepatocytes resulted in suppressed ID1 expression and connective tissue growth factor (CTGF) secretion, subsequently activating the p38 and p65 signaling pathways in HSCs and thereby alleviating liver fibrosis [158]. Since ID1 is associated with HCC development, this suggests that an elevated expression of ID1 may play a significant role in the early stages of hepatocarcinogenesis in patients with liver cirrhosis [159]. Therefore, further studies are needed to investigate the role of the BMP9–ID1 axis in liver fibrosis. Besides ID1, TGF-β1 also promotes the activities of several signaling pathways including Smad2/3, TAK1, JNK, p38, MAPKs, RhoA–ROCK, JAK–STAT3, and PI3K–AKT, which contribute to the induction of hepatic fibrosis [160].

## 8. BMP9 and Portopulmonary Hypertension

Portopulmonary hypertension (PoPH) is a form of PAH that occurs in individuals with portal hypertension and liver cirrhosis and is characterized by increased blood pressure in the portal vein responsible for carrying blood from the digestive organs to the liver. In PoPH, the elevated pressure within the portal system induces changes in the pulmonary blood vessels, resulting in their constriction and reduced blood flow. Consequently, this leads to increased pressure in the pulmonary arteries, ultimately causing PAH [161,162]. Recent studies have highlighted the crucial roles of BMP9 in PoPH [163]. Reduced levels of circulating BMP10 and BMP9, along with elevated levels of endoglin, have been associated with disease severity, decompensation, and pulmonary vascular syndromes in patients with cirrhosis [164]. Circulating levels of BMP9 were found to be decreased in PoPH patients compared to healthy individuals or those with other etiologies of PAH, suggesting BMP9 as a potential biomarker for PoPH [165]. Additionally, studies have shown decreased levels of both BMP9 and BMP10 in plasma samples and liver specimens from patients with decompensated cirrhosis, including those with hepatopulmonary syndrome (HPS) or PoPH. Concurrently, the increased expression of endoglin mRNA was observed in cirrhotic livers, and elevated levels of circulating sEng in PoPH and HPS patients indicated enhanced endothelial sEng shedding in these conditions [161,164,166]. Animal models have demonstrated that a deficiency of BMP9 exacerbates PoPH, while the protective effect of BMP9 in PoPH has been attributed to its interaction with ALK1 and endoglin in pulmonary vascular endothelial cells [165]. These findings suggest that BMP9 is a sensitive and specific biomarker of PoPH, predicting transplant-free survival and the presence of PAH in liver disease.

## 9. BMP9 and Hepatopulmonary Syndrome

Hepatopulmonary syndrome (HPS) is a condition that affects the liver, lungs, and circulation. It is characterized by the presence of liver disease, abnormalities in arterial oxygenation, and the development of intrapulmonary vascular dilations called pulmonary arteriovenous malformations (PAVMs). Hereditary hemorrhagic telangiectasia is associated with approximately 80% of PAVMs [167]. Considering this, there has been speculation about the association between BMP9 and HPS. Some studies have reported that Krüppel-like factor 6 (KLF6) mediates pulmonary angiogenesis in an experimental rat model of hepatopulmonary syndrome and is exacerbated by BMP9 [168]. Also, research on a rat model showed that elevated circulating BMP9, secreted from the liver, promotes pulmonary angiogenesis in HPS rats via the ALK1–endoglin–Smad1/5/9 pathway [169]. And another study including HPS patients and an animal model showed that portal hypertension-induced loss of BMP9 signaling contributes to HPS development [170]. However, it is worth noting that some researchers believe that the evidence linking BMP9 to HPS is insufficient, and further investigation is required to establish a clear relationship between BMP9 and HPS [125,171]. Therefore, more research is needed to fully elucidate the role of BMP9 in HPS and provide stronger evidence for its involvement.

## 10. BMP9 and HCC

HCC is the most prevalent form of liver cancer and is associated with a poor prognosis. In recent decades, there has been a consistent increase in the incidence of HCC and the number of deaths caused by this disease. Although BMP9 has been implicated in cancers, its precise role in the development and progression of HCC remains unclear [172]. Previous studies have reported that BMP9 is elevated in approximately 40% of HCC tissues [172,173], while our own previous study showed an even higher percentage at nearly 70% [174]. However, data from the TCGA-LIHC database suggests that the expression level of BMP9 is lower in HCC compared to normal liver tissue. This discrepancy might be attributed to different etiologies and subtypes of liver cancer, which can have varying molecular characteristics and BMP9 expression patterns. Further research is needed to better understand the specific contexts in which BMP9 is involved in HCC and its implications for diagnosis, treatment, and prognosis.

The process of EMT involves a series of steps in which epithelial cells undergo temporary de-differentiation, resulting in a change in their plasticity and the adoption of a mesenchymal phenotype. EMT plays a crucial role in the progression of carcinoma, particularly in cancer invasion and metastasis. During EMT, cells lose their intercellular contacts, primarily due to the downregulation of E-cadherin, leading to increased motility and the ability to spread into nearby or distant tissues. Epithelial plasticity has gained significant attention in HCC. Several families of transcription factors, such as the SNAI family (including Snail (SNAI1) and Slug (SNAI2)), Twist family (Twist1, Twist2, E12, E47, and IDs), and ZEB family (ZEB1 and ZEB2), play important roles in driving the progression of EMT [175]. In HCC, it has been observed that BMP9 expression is associated with increased Smad1 phosphorylation, elevated levels of N-cadherin and Snail, and decreased levels of E-cadherin. Treatment with BMP9 at a concentration from 10 to 50 ng/mL induced cell migration, morphological conversion from epithelial to fibroblastic cells, and the upregulation of the mesenchymal marker Vimentin while reducing the expression of E-cadherin in the HCC cell lines HLE and HepG2 [49,176]. These findings suggest that BMP9 may promote EMT in HCC, contributing to the acquisition of a more invasive and migratory phenotype by HCC cells. In contrast to its promotion of EMT in HCC, BMP9 inhibits EMT in hepatocytes. The opposing effects of BMP9 could be attributed to the distinct cellular characteristics between hepatocytes (non-malignant) and HCC cells (malignant), as well as the differential activation of signaling pathways within each cell type [35].

MAPKs are a family of kinases that play a critical role in various cancer processes [177,178]. Among them, the p38–MAPK pathway is particularly important in regulating cell cycle progression by influencing the transcriptional machinery. The activation of the p38 MAPK pathway has been found to inhibit HCC cell proliferation and promote apoptosis [179,180]. Importantly, the p38–MAPK pathway represents a Smad-independent signaling mechanism that can be activated by BMPs [71,181]. It has been observed that p38 MAPK activation is essential for the protective effect of BMP9 against serum detoxification-induced apoptosis, and its involvement in promoting HepG2 cell growth has also been noted [173]. These findings highlight the significance of the p38–MAPK pathway in mediating the effects of BMP9, indicating its potential role in modulating cell proliferation and apoptosis in HCC cells.

BMP9 has the ability to induce cancer stem cell properties in a specific subtype of liver cancer stem cells (LCSCs) that are characterized by the presence of an epithelial cell adhesion molecule (EpCAM). The Wnt/β-catenin signaling pathway has been extensively associated with various human cancers. Abnormal activation of this pathway is closely linked to increased cancer prevalence, malignant progression, poor prognostics, and even elevated cancer-related mortality rates [182,183]. C-myc, an oncogene, is identified as a downstream target of the Wnt/β-catenin signaling pathway [184,185]. Patients with a high expression of BMP9 in liver cancer tissues tend to have a poorer prognosis. It has been observed that BMP9 can activate the Wnt/β-catenin signaling pathway through ID1, leading to the upregulation of C-myc expression. This activation ultimately induces cancer stem cell properties in cell lines containing EpCAM-positive LCSCs. To counteract these effects, the application of BMP receptor inhibitors, specifically K02288 and LDN-212854, has shown effectiveness in diminishing the cancer stem cell properties induced by BMP9 in EpCAM-positive HCC cells [174]. Another study has also revealed that BMP9 can inhibit m^6^A methylation modification and promote cell cycle progression in HCC cells. BMP9 is capable of inducing the expression of fat mass and obesity-associated protein (FTO) while reducing the expression of YTH N6-methyladenosine RNA binding protein F2 (YTHDF2). This leads to decreases in global RNA m^6^A methylation in HCC and m^6^A methylation within the 5‘-UTR regions of CyclinD1 mRNA. Consequently, the increased expression of CyclinD1 facilitates cell cycle progression in HCC cells [186].

HCC is a highly vascularized solid tumor, and angiogenesis plays a crucial role in its development [187,188]. The HIF-1α/VEGFA signaling pathway is widely implicated in the occurrence, progression, and prognosis of HCC [189,190]. It has been observed that BMP9-ID1 signaling is correlated with HIF-1α expression in HCC tissues. BMP9 can induce the activation of HIF-1α/VEGFA signaling, promoting the secretion of VEGFA, which leads to lumen formation in HUVECs (human umbilical vein endothelial cells). Furthermore, the application of BMP receptor inhibitors can diminish the activation of HIF-1α/VEGFA signaling induced by BMP9, as well as the secretion of VEGFA and lumen formation in HUVECs [191]. Another study conversely demonstrated that BMP9 could induce vascular normalization in hepatitis B virus-associated HCC. In patients with HBV-related HCC, reduced BMP9 expression caused vascular abnormalities that hindered intra-tumoral cytotoxic lymphocyte infiltration. The overexpression of BMP9 in HBV-infected HCC cells facilitated cytotoxic lymphocyte infiltration through vascular normalization. This effect was achieved by inhibiting the Rho–ROCK–myosin light chain (MLC) signaling cascade, ultimately improving the efficacy of immunotherapy. Additionally, ultrasound-targeted microbubble destruction (UTMD)-mediated BMP9 delivery showed therapeutic efficacy when combined with a programmed death-ligand 1 (PD-L1) antibody in HepG2.2.15 cell xenografts in immune-deficient mice [192].

These findings suggest that the influence of BMP9 signaling on HCC is complicated. Further research is needed to elucidate the mechanisms through which BMP9 signaling regulates HCC derived from different etiologies.

## 11. Summary and Future Expectations

**Figure 5:**
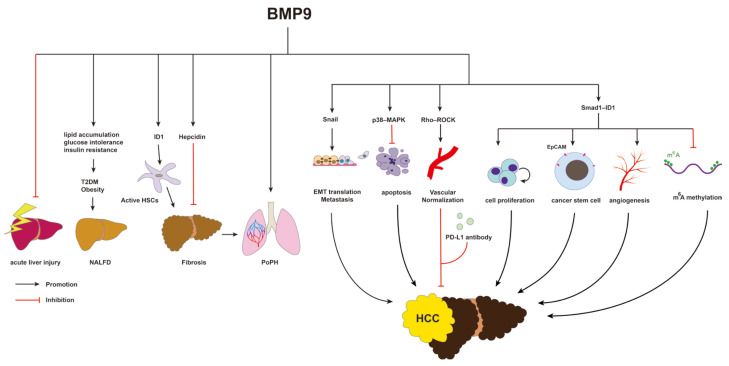
Schematic diagram of the effects of BMP9 on liver diseases.

BMP9 is a multifunctional cytokine that plays a crucial role in various cellular processes, including bone and cartilage formation, glucose and lipid metabolisms, iron balance, neuron differentiation, angiogenesis, and lymphangiogenesis. The liver serves as the primary organ for BMP9 expression and secretion, indicating its potential involvement in the progression of liver diseases [193]. In this review, we have summarized the functions and mechanisms of BMP9 in diverse liver diseases, including viral hepatitis, acute liver injury, NAFLD, hepatic fibrosis, PoPH, HPS, and HCC (Figure 5). It has been observed that BMP9 is downregulated in cases of HCV infection and acute liver injury. Moreover, BMP9 has shown beneficial effects on two risk factors for NAFLD and obesity. Its effects include regulating glucose and lipid metabolisms by inhibiting liver gluconeogenesis, promoting the transformation of WAT to BAT, enhancing insulin sensitivity, and inhibiting liver lipid deposition. Regarding liver fibrosis, BMP9 has the potential to induce its progression through the activation of the ALK1–Smad1/5 signaling pathway and associated targets such as ID1, Hepcidin and Snail. However, the co-receptor endoglin exerts a protective effect against liver fibrosis through the ALK5–Smad2/3 signaling pathway. In the case of PoPH, decreased BMP9 levels suggest a possible protective effect in this condition. The role of BMP9 in HPS remains unclear, and further research is required to understand its specific involvement. In HCC, BMP9 has been found to induce the EMT process and upregulate the expression of EMT markers such as Vimentin while reducing E-cadherin in HCC cell lines. Additionally, BMP9 promotes HCC cell proliferation through the activation of both the Smad1/5–ID1 signaling pathway and the p38–MAPK signaling pathway. Moreover, BMP9 enhances the cancer stem cell properties in Huh7 and MT cell lines characterized by EpCAM positive LCSCs. Furthermore, BMP9 induces angiogenesis by activating the HIF-1α/VEGFA signaling pathway in the Huh7 and MT cell lines. In HBV-related HCC, it has been observed that HBV infection reduces the expression of BMP9. However, BMP9 treatment can induce vascular normalization and facilitate the infiltration of cytotoxic lymphocytes through the Rho–ROCK–MLC signaling pathway. These findings suggest potential therapeutic efficacy when combining BMP9 with a PD-L1 antibody for treating HBV-related HCC.

In addition to BMP9, ALK1 is also a promising therapeutic target for the treatment of HCC. Currently, there are three drugs under development targeting ALK1. They are the monoclonal antibody Ascrinvacumab, the bispecific antibody ALK1/VEGF, and the monoclonal antibody TATX-21. Previously, there was also a fusion protein called Dalantercept, but it showed limited efficacy in HCC [194]. These three investigational drugs are at different stages of development. Ascrinvacumab has entered clinical trials, while the other two drugs are still in early stage research. These drugs have different therapeutic areas. Ascrinvacumab mainly targets solid tumors, including HCC [195,196], and TATX-21 could be used for the treatment of arterial sclerosis and heart disease. In summary, as a novel target for solid tumors and cardiovascular diseases, ALK1 holds promising potential for breakthroughs.

In conclusion, BMP9 plays a dual role depending on specific conditions and diseases. A thorough comprehension of BMP9 will significantly enhance our understanding of viral hepatitis, acute liver injury, obesity, T2DM, NAFLD, liver fibrosis or cirrhosis, PoPH, and HCC, potentially establishing it as a valuable biomarker for diagnosing and monitoring various liver diseases. However, more evidence is needed to support the therapeutic potential of the BMP9-ALK1 axis for HCC treatment.

## Figures and Tables

**Table 1:** The roles of BMP9 in NALFD.

Molecular Mechanism	Effects of the Pathway	Effects of BMP9	Study Type	Reference
BMP9 promotes M1 macrophage polarization	BMP9 enhances NF-κB signaling	BMP9 promotes nonalcoholic steatohepatitis	Animal model	Jiang (2021) [111,125]
BMP9 reduces obesity, improves glucose metabolism, alleviates hepatic steatosis, and decreases inflammation	BMP9 decreases the promoter chromatin accessibility of *CERS6*, *FABP4*, *FOS*, and *TLR1*	BMP9 suppresses NALFD	Animal model	Sun (2021) [104]
FINS, LDL-C, HDL-C, and BMI were independent factors impacting serum BMP9 levels	Serum levels of BMP9 are significantly lower in patients with T2DM and NAFLD	BMP9 is an independent risk factor for patients with T2DM and NAFLD	Cross-sectional study	Hao (2022) [98]
Alcohol induces the activation of BMP signaling	Alcohol induces the expression of BMP2, BMP4, BMP7, BMP9, Smad1, and Smad4	BMP signaling exerts anti- and/or pro-fibrotic effects	Animal model	Hong (2023) [126]
BMP9 attenuates triglyceride accumulation by enhancing PPARα promoter activity via the activation of pSmads	BMP9 attenuates triglyceride accumulation by enhancing PPARα promoter activity via the activation of pSmads	Loss of BMP9 promotes liver steatosis	Cell line and animal model	Yang (2020) [105]
BMP9 decreases ALT as well as cholesterol and enhances brown adipogenesis	BMP9 promotes UCP1 expression through the activation of FGF21	BMP9 suppresses NALFD and obesity	Animal model	Kim (2016) [103]
There is a positive correlation between BMP9 levels and NASH/NAFLD	Patients with BMP9 > 1188 pg/mL show worse disease in NASH/NAFLD	BMP9 can be biomarker for NASH/NAFLD	Cross-sectional study	Bocci (2022) [96]
Hepatic BMP9 expression negatively correlates with steatosis in obese patients with diabetes	BMP9 directly induces FGF19 in gut	Under pre-steatotic conditions, it is likely that increased levels of BMP9 would have beneficial effects	Cross-sectional study	Drexler (2023) [100]
BMP9 is negatively associated with WHR, FBG, 2h-OGTT, HbA1c, TG levels, and HOMA-IR; on the other hand, BMP9 is positively associated with FFA and HDL levels	Circulating BMP9 is negatively associated with metabolic syndrome and insulin resistance	BMP9 is independently associated with T2DM, HOMA-IR, and FFA	Cross-sectional study	Xu (2017) [118]
BMP9 level is negatively correlated with HbA1c, FBG, OGTT, AUC glucose, and HOMA-IR	Circulating BMP9 levels are significantly lower in newly diagnosed T2DM patients compared to healthy people	The decreased levels of circulating BMP9 could serve as a marker of insulin resistance in T2DM patients	Cross-sectional study	Luo (2017) [119]
	Platycodin induces BMP9 expression and reduces Smad4 expression	BMP9 rectifies blood glucose and lipid metabolism disorders	Animal model	Luan (2014) [120]
The deletion of BMP9 elevates the PEPCK protein levels and reduces the levels of InsR and Akt phosphorylation		The deletion of BMP9 exacerbates insulin resistance and glucose intolerance in mouse models fed a high-fat diet	Animal model	Jia (2019) [121]

CERS6, Ceramide synthase 6; FABP4, Fatty Acid Binding Protein 4; TLR1, Toll Like Receptor 1; NAFLD, nonalcoholic fatty liver disease; FINS, fasting insulin; LDL-C, low-density lipoprotein cholesterol; HDL-C, high-density lipoprotein cholesterol; BMI, body mass index; T2DM, type 2 diabetes mellitus; UCP1, uncoupling protein 1; FGF21, fibroblast growth factor 21; WHR, waist–hip ratio; FBG, fasting blood glucose; 2h-OGTT, 2 h blood glucose after glucose overload; TG, triglyceride; HOMA-IR, homoeostasis model assessment of insulin resistance; FFA, free fatty acid; AUC glucose, the area under the curve for glucose; PEPCK, phosphoenolpyruvate carboxykinase; InsR, insulin receptor.

## Abbreviations

α-SMA	alpha smooth muscle actin
APAP	acetaminophen
ALI	acute liver injury
ALP	alkaline phosphatase
AIH	autoimmune hepatitis
AUC	the area under the curve for glucose
BAT	brown adipose tissue
BMI	body mass index
BMPs	bone morphogenetic proteins
CBP	CREB-binding protein
CCl4	carbon tetrachloride
C/EBPα	CCAAT/enhancer binding protein (C/EBP) alpha
CHB	chronic hepatitis B
CREB	cAMP response-element binding protein
CTGF	connective tissue growth factor
DDC	3,5-diethoxicarbonyl-1,4-dihydrocollidine
5-diethoxicarbonyl-1	4-dihydrocollidine
ECM	extracellular matrix
EGF	epidermal growth factor
EMT	epithelial-to-mesenchymal transition
ENG	endoglin
EpCAM	epithelial cell adhesion molecule
ERK	extracellular signal-regulated kinase
FBG	fasting blood glucose
FFA	free fatty acid
FFC	high fat, fructose, and cholesterol diet
FGF	fibroblast growth factor
FINS	fasting insulin
FTO	fat mass and obesity-associated protein
GDF	growth differentiation factor
GSK3	glycogen synthase kinase 3
HAI	histological activity index
HCC	hepatocellular carcinoma
HCV	hepatitis C virus
HDL-C	high-density lipoprotein cholesterol
HGF	hepatocyte growth factor
HHT	hereditary hemorrhagic telangiectasia
HIF-1α	hypoxia inducible factor 1 subunit alpha
HOMA-IR	homoeostasis model assessment of insulin resistance
HPS	hepatopulmonary syndrome
HSC	hepatic stellate cell
HUVEC	human umbilical vein endothelial cell
ID1/2/3	inhibitor of differentiation-1/2/3
IFN	interferon
IL-8	interleukin-8
InsR	insulin receptor
IR	insulin resistance
IRF	interferon regulatory factors
JNK	c-Jun N-terminal kinases
KC	Kupffer cells
KLF6	Krüppel-like factor 6
LDL-C	low-density lipoprotein cholesterol
LIMK	LIM Domain Kinase
LPS	lipopolysaccharide
LSEC	liver sinusoidal endothelial cell
LCSC	liver cancer stem cell
MAPK	mitogen-activated protein kinases
MB109	recombinant derivative of human BMP9
MLC	myosin light chain
MMP-7	matrix metalloproteinase-7
NAFLD	nonalcoholic fatty liver disease
NASH	nonalcoholic steatohepatitis
OGTT	oral glucose tolerance tests
PAH	pulmonary arterial hypertension
PAVM	pulmonary arteriovenous malformations
PBC/PSC	primary biliary cholangitis/primary sclerosing cholangitis
PD-L1	programmed death-ligand 1
PEPCK	phosphoenolpyruvate carboxykinase
PGC1α	proliferator-activated receptor-gamma coactivator 1 alpha
PI3K	Phosphoinositide 3-kinases
PoPH	portopulmonary hypertension
PPARα	peroxisome proliferator-activated receptor α
ROCK	Rho-Associated Coiled-Coil-Containing Protein Kinase
TAG	triglyceride
TGF-β	transforming growth factor β
T2DM	type 2 diabetes mellitus
UCP1	uncoupling protein 1
UDCA	Ursodesoxycholic acid
USP18	Ubiquitin specific peptidase 18
UTMD	ultrasound-targeted microbubble destruction
VEGF	vascular endothelial growth factor
WAT	white adipose tissue
WHR	Waist-hip ratio
WNT2	Wnt Family Member 2
YTHDF2	YTH N6-methyladenosine RNA binding protein F2
ZEB	zinc finger E-box binding
